# *Bacillus subtilis* as a host for mosquitocidal toxins production

**DOI:** 10.1111/1751-7915.13648

**Published:** 2020-08-30

**Authors:** Emanuela Ursino, Alessandra M. Albertini, Giulia Fiorentino, Paolo Gabrieli, Viola Camilla Scoffone, Angelica Pellegrini, Giuliano Gasperi, Alessandro Di Cosimo, Giulia Barbieri

**Affiliations:** ^1^ Department of Biology and Biotechnology Università degli Studi di Pavia Pavia Italy; ^2^ Present address: Department of Biosciences Università degli Studi di Milano Milano Italy

## Abstract

*Aedes albopictus* transmits several arboviral infections. In the absence of vaccines, control of mosquito populations is the only strategy to prevent vector‐borne diseases. As part of the search for novel, biological and environmentally friendly strategies for vector control, the isolation of new bacterial species with mosquitocidal activity represents a promising approach. However, new bacterial isolates may be difficult to grow and genetically manipulate. To overcome these limits, here we set up a system allowing the expression of mosquitocidal bacterial toxins in the well‐known genetic background of *Bacillus subtilis*. As a proof of this concept, the ability of *B. subtilis* to express individual or combinations of toxins of *Bacillus thuringiensis israelensis* (Bti) was studied. Different expression systems in which toxin gene expression was driven by IPTG‐inducible, auto‐inducible or toxin gene‐specific promoters were developed. The larvicidal activity of the resulting *B. subtilis* strains against second‐instar *Ae. albopictus* larvae allowed studying the activity of individual toxins or the synergistic interaction among Cry and Cyt toxins. The expression systems here presented lay the foundation for a better improved system to be used in the future to characterize the larvicidal activity of toxin genes from new environmental isolates.

## Graphical abstract

We investigated the feasibility of using *B. subtilis* as host to produce known and yet unknown mosquitocidal bacterial toxins. As a proof of this concept, by using different expression systems, the ability of *B. subtilis* to express individual or combinations of toxins of *Bacillus thuringiensis israelensis* (Bti) was studied. The toxicity of the obtained recombinant strains against *Ae. albopictus* larvae was assessed.

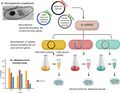

## Introduction

Mosquito‐borne viruses and pathogens are responsible for the deadliest diseases, causing more than 700 000 deaths each year (Gates, 2017). Given that no vaccines or drugs are available to prevent or cure the majority of them, the most effective control strategy is targeting the mosquito vectors through the use of insecticides. However, the spread of populations resistant to their action is hampering their efficacy (Moyes *et al*., 2017) and the environmental pollution associated with their extensive use is raising concerns (Benelli and Beier, 2017). Therefore, the development of new, possibly environmentally friendly tools is urgently needed. In this context, the biological control of mosquitoes using bacterial toxins, such as those produced by *Bacillus thuringiensis israelensis*, is increasingly attracting attention as a possible alternative control strategy in many field settings and the expansion of the toolbox of available toxins active against mosquitoes is desirable (Contreras *et al*., 2019). The isolation of new bacterial species with mosquitocidal activity is an active research topic (Ramirez *et al*., 2014). In order to identify and characterize new toxins from newly isolated mosquitocidal bacteria, it would be useful to create systems allowing the expression of the genes encoding putative new toxins in a well‐known genetic background. This strategy would allow investigating the structural and functional properties of the protein of interest using well‐studied organisms that are known to be safe for human health, overcoming the limitations of working with environmental isolates that could be difficult to genetically manipulate and possibly toxic for human beings.

On these premises, the specific aim of this work is the set‐up in *Bacillus subtilis* of expression systems that can be used to produce and characterize mosquitocidal toxins of new environmental isolates. As a proof of concept, we created *Bacillus subtilis* strains expressing one or more toxin genes of *Bacillus thuringiensis israelensis* (Bti) and tested their toxicity towards larvae of the Asian tiger mosquito, *Aedes albopictus*.

According to the Global Invasive Species Database (2019), *Ae. albopictus* is one of the world’s worst invasive species and is a competent vector of many arboviruses, including West Nile, Yellow fever, Dengue, Zika and Chikungunya (Benedict *et al*., 2007). It is native to Southeast Asia but human activities, especially the international trade in flowers/plants or used car tires, favoured by global warming, allowed it to spread to all continents, except Antarctica (Kraemer *et al*., 2015).

*Bacillus subtilis* is a well‐known and easy‐to‐handle Gram‐positive soil bacterium that is considered as a GRAS organism (Generally Recognized As Safe). Its ability to produce and secrete high concentrations of proteins into the medium (Harwood, 1992) renders it an efficient expression host for the production of proteins of industrial interest (Schallmey *et al*., 2004; Westers *et al*., 2004). Two major extracellular proteases (AprE – also called subtilisin – and NprE) are expressed at the beginning of the stationary phase. Because of their commercial interest, they have been extensively studied, leading to a good knowledge of their synthesis and regulation systems. In particular, the expression of *aprE* is controlled by many different transcription factors that can bind different sites in the *aprE* regulatory region, enhancing or lowering its expression (Ogura *et al*., 2004). A major positive regulator of *aprE* expression is DegU. This protein is part of the DegS‐DegU two‐component system. In its phosphorylated form, DegU acts as an activator of *aprE* (Kunst *et al*., 1974). A particular mutation of the *degU* gene, *degU32(hy)*, mimics the phosphorylated, active form of the transcriptional activator DegU (DegU‐P), enhancing the transcription of *aprE* (Mader *et al*., 2002). In this work, the knowledge of the mechanisms controlling gene expression in *B. subtilis* was exploited to express Bti toxins.

The larvicidal activity of Bti is a long and well‐known biological phenomenon and it is due to the presence of pBtoxis, a 128 kb plasmid, which encodes four major protoxins (Cry4Aa, Cry4Ba, Cry11Aa and Cyt1Aa) and two minor toxins (Cry10Aa and Cyt2Ba) that are expressed as parasporal crystalline bodies at the onset of sporulation (Ben‐Dov *et al*., 1999; Berry *et al*., 2002). This plasmid also contains additional genes that encode for proteins that help the folding and activity of the toxins Cry11Aa and Cyt1Aa. Among the Dipteran‐specific toxins synthesized by Bti in late stationary phase during sporulation, the Cry11Aa δ‐endotoxin targets very efficiently *Ae. albopictus* larvae (Otieno‐Ayayo *et al*., 2008; Ben‐Dov, 2014). The gene encoding the Cry11Aa toxin is included in the *p19‐cry11Aa‐p20* tri‐cistronic operon (Berry *et al*., 2002). While the function of P19 is not known, the P20 protein was previously reported to stabilize both Cyt1Aa and Cry11Aa by protecting the nascent polypeptides from proteolysis (Adams *et al*., 1989; Visick and Whiteley, 1991; Xu *et al*., 2001; Sazhenskiy *et al*., 2010) and to enhance the expression of Cry11Aa not only in *B. thuringiensis* (Wu and Federici, 1995) but even in recombinant *E. coli* (Xu *et al*., 2001).

The deep knowledge of the *B. subtilis* genetics and of the *B. thuringensis israeliensis* toxins allowed us to investigate the feasibility of using *B. subtilis* as host to produce known and yet unknown larvicidal toxins in a safe organism.

## Results

### Expression of Cry11Aa by an IPTG‐inducible system

To evaluate the ability of *B. subtilis* as an expression system for Bti δ‐endotoxins, the *cry11Aa* gene, with and without its downstream gene *p20*, was amplified from a wild‐type strain of *Bacillus thuringiensis israelensis* (4Q1) and cloned into the *amyE* chromosomal integrative plasmid pDR111 (Ben‐Yehuda *et al*., 2003), downstream of the *Phyperspank* IPTG‐inducible promoter. The obtained constructs were inserted by double cross‐over in the *amyE* gene of *Bacillus subtilis* strain PB1831. The resulting strains PB7223 (*amyE*::*Phyperspank*‐*cry11Aa*) and PB7226 (*amyE*::*Phyperspank*‐*cry11Aa*‐*p20*; Fig. S1A) displayed no growth defects compared to the control strain PB7222 (*amyE*::*spc*) when grown in 2xSG sporulation medium (Fig. S1B).

As revealed by SDS‐PAGE analysis on cells collected at 20 h after IPTG induction, Cry11Aa was successfully expressed by both strains but, unexpectedly, the amount of Cry11Aa produced by *B. subtilis* cells expressing both *cry11Aa* and *p20* resulted to be about 15‐fold lower than that displayed by cells expressing only *cry11Aa* (Fig. S1C). Notably, P20 expression could not be detected by SDS‐PAGE analysis even in the control sample Bti 4Q1.

**Fig. 1:**
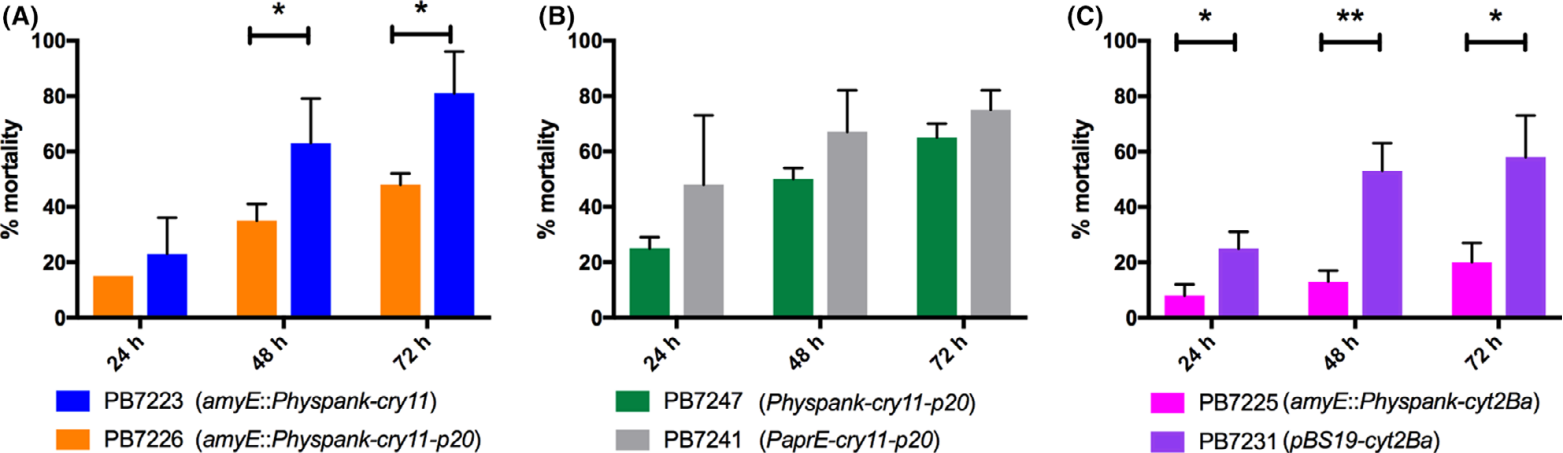
Percentage of *Aedes albopictus* larval mortality at different time points after treatment with 2 g l^−1^ (wet weight) of cells, spore–parasporal bodies mixture (collected at T20) of strains A. PB7223, PB7226, B. PB7241, PB7247, C. PB7225, PB7231. *P < 0.05; **P < 0.01. Results are means ± SD of at least three independent experiments, each performed in duplicate.

Accordingly, strain PB7223, expressing only Cry11Aa, displayed a higher larvicidal activity against *Aedes albopictus* as compared to PB7226, expressing both proteins (48‐h, *P = *0.033; 72‐h, *P = *0.016; Fig. 1A). The larvicidal activity induced by both strains increased over time, up to 72 h of exposure to the toxins. At 96 h after the beginning of the assay, no significant increase in mortality was detected compared to the 72‐h time point (data not shown).

Previous studies reported that the P20 helper protein is required for efficient expression of Cyt1Aa and other Cry toxins (Wu and Federici, 1993; Sazhenskiy *et al*., 2010). As in this work we are interested in expressing different combinations of genes and in studying the interaction among different toxins of Bti, the *p20* gene was maintained in the construct.

### Expression of Cry11Aa and the helper protein P20 in an auto‐inducible system

In order to create an auto‐inducible expression system, the region upstream of the *B. subtilis aprE* gene, extending from positions −614 to −1 with respect to the beginning of the *aprE* coding sequence and comprising the entire transcriptional control region (TCR) and the 5’UTR of the gene (Ferrari *et al*., 1993; Ogura *et al*., 2004), was fused to the Bti *cry11Aa‐p20* genes. The obtained construct was cloned into the *B. subtilis* integrative plasmid pJM113 (Perego, 1993). The resulting pBG105 vector and the control vector pBG109, bearing only the TCR and 5’UTR of *aprE*, were integrated by single cross‐over at the *PaprE* region of the *B. subtilis* wild‐type strain PB168. The resulting strains did not show detectable levels of Cry11Aa by SDS‐PAGE and did not have any larvicidal activity towards *Ae. albopictus* larvae (data not shown). In order to upregulate *PaprE* activity and to increase Cry11Aa protein yield, pBG105 and pBG109 were used to transform PB7007, a strain carrying the *degU32(hy)* allele and the inactivation (deletion of part of the cds) of the two major exoprotease genes *aprE* and *nprE*, to allow a more stable production of heterologous proteins. The resulting strains PB7241 (Δ*aprE*::*PaprE‐cry11Aa‐p20*) and PB7242 (Δ*aprE*::*PaprE*; Fig. S2A) displayed similar growth patterns in 2xSG sporulation medium (Fig. S2B). SDS‐PAGE analysis of the cells, spore and parasporal body mixtures collected at 4, 15, 20, 24, 48 and 72 h after the beginning of the stationary phase showed that the expression of the heterologous toxin gene starts at the onset of sporulation, concomitantly with the activation of the *aprE* promoter. The production of Cry11Aa is still absent 4 h after the beginning of the stationary phase, while it accumulates up to 72 h (Fig. S2C). Treatment with 2 g l^−1^ of spore–parasporal bodies mixtures of the auto‐inducible strain PB7241 collected 20 h after the beginning of stationary phase (T20) induced almost 80% mortality in *Ae. albopictus* larvae after 72h of treatment (Fig. 1B).

To compare the expression and the toxicity of the above‐mentioned *Phyperspank‐cry11Aa‐p20* and *PaprE‐cry11Aa‐p20* constructs in the same genetic background, the *Phyperspank‐cry11Aa‐p20* construct was integrated at the *amyE* locus of the *degU32(hy) Bacillus subtilis* strain PB7007. The resulting strain (PB7247) did not show any growth defect but expressed Cry11Aa at a lower level compared to PB7241 (Fig. S3A and B). Accordingly, when assayed for toxicity, at all sampling times studied, PB7247 spores–parasporal bodies conferred a lower larval mortality (not statistically significant, *P = *0.32) compared to that of the strain carrying the *PaprE*‐dependent auto‐inducible construct (PB7241; Fig. 1B). Interestingly, expression of the *Phyperspank‐cry11Aa‐p20* construct in a *degUhy* background (PB7247) confers a higher toxicity against *Ae. albopictus* larvae compared to PB7226 strain (72‐h time point, *P = *0.0003; Fig. 1A and B).

### Cyt1Aa is toxic against *B. subtilis*

Previous studies proposed a synergist effect of Cyt1Aa on Cry11Aa (Perez *et al*., 2005; Perez *et al*., 2007). We therefore analysed the toxicity of *B. subtilis* strains expressing Cyt1Aa. First, the pBtoxis region encompassing the *cyt1Aa* and *p21* genes was cloned into pDR111, under the control of the *Phyperspank* promoter. The gene *p21* is oriented in the opposite direction with respect to *cyt1Aa* and cannot be expressed by our construct. However, this sequence was included in the cloned region as it comprises the *cyt1Aa* terminator hairpin which plays a role in *cyt1Aa* mRNA stability (Sakano *et al*., 2017). The resulting *Phyperspank*‐*cyt1Aa‐p21* construct was integrated in the *amyE* gene of PB1831. Surprisingly, Cyt1Aa could not be detected by SDS‐PAGE analysis of PB7232 cells–spores mixture collected at 72h after induction, suggesting that single copy integration of *cyt1Aa* in the *B. subtilis* chromosome under the control of the IPTG‐inducible promoter is not sufficient for efficient Cyt1Aa expression (Fig. S4). The *cyt1Aa‐p21* gene cluster was then cloned under the control of the *cyt1Aa* promoter region into the *B. subtilis* multicopy replicative plasmid pBS19. The PB1831 derivative strain carrying the pBS19‐*Pcyt1Aa‐p21* plasmid (PB7230) produced Cyt1Aa protein (Fig. S4) and caused 85% mortality against *Aedes albopictus* larvae after 24 h from the beginning of the assay (data not shown). Unfortunately, Cyt1Aa overexpression in our *B. subtilis* strain is toxic, inhibiting growth, cell division and sporulation hence leading to the death of the host cells (Table S3; Fig. S5). For this reason, we decided to focus our attention on another cytolytic protein: the minor toxin Cyt2Ba.

### Coexpression of Cry11Aa and Cyt2Ba toxins

In order to study the interaction between Cyt2Ba and Cry11Aa toxins, the gene encoding the cytolytic protein Cyt2Ba was cloned downstream of the *Phyperspank* promoter in plasmid pDR111 and the resulting construct was integrated by double cross‐over in the *amyE* gene of *B. subtilis* PB1831 (strain PB7225). The *cyt2Ba* gene was also cloned in the pBS19 multicopy replicative plasmid under the control of its own promoter (strain PB7231). The two resulting recombinant strains did not show any growth defect compared to their respective control strains PB7222 (*amyE::spc*) and PB7229 (pBS19) respectively (Fig. S6A). SDS‐PAGE analysis of the pellets collected 24 h after the beginning of the stationary phase showed a lower level of Cyt2Ba expression in strain PB7225 compared to PB7231 (Fig. S6B). Accordingly, when assayed for toxicity, PB7231 displayed a significantly higher larvicidal activity against *Aedes albopictus* compared to PB7225 (24‐h, *P* = 0.029; 48‐h, *P = *0.008; 72‐h, *P = *0.030; Fig. 1C).

To evaluate whether coexpression of Cry11Aa and Cyt2Ba resulted in a synergistic or additive effect in terms of larvicidal activity, two additional strains were prepared. Strain PB7240 was obtained by transformation of the PB1831 derivative carrying the *amyE::Phyperspank*‐*cry11p20* construct (PB7226) with plasmid pBS19‐*Pcyt2Ba*. Strain PB7265 was prepared by integrating the *Phyperspank*‐*cyt2Ba* construct in the *amyE* gene of the *degU32(hy)* strain PB7241, carrying the *PaprE‐cry11Aa‐p20* construct integrated by single cross‐over in the *aprE* locus. Coexpression of the two toxins did not affect the growth characteristics of the two strains compared to the corresponding parental strains (Fig. S7).

**Fig. 2:**
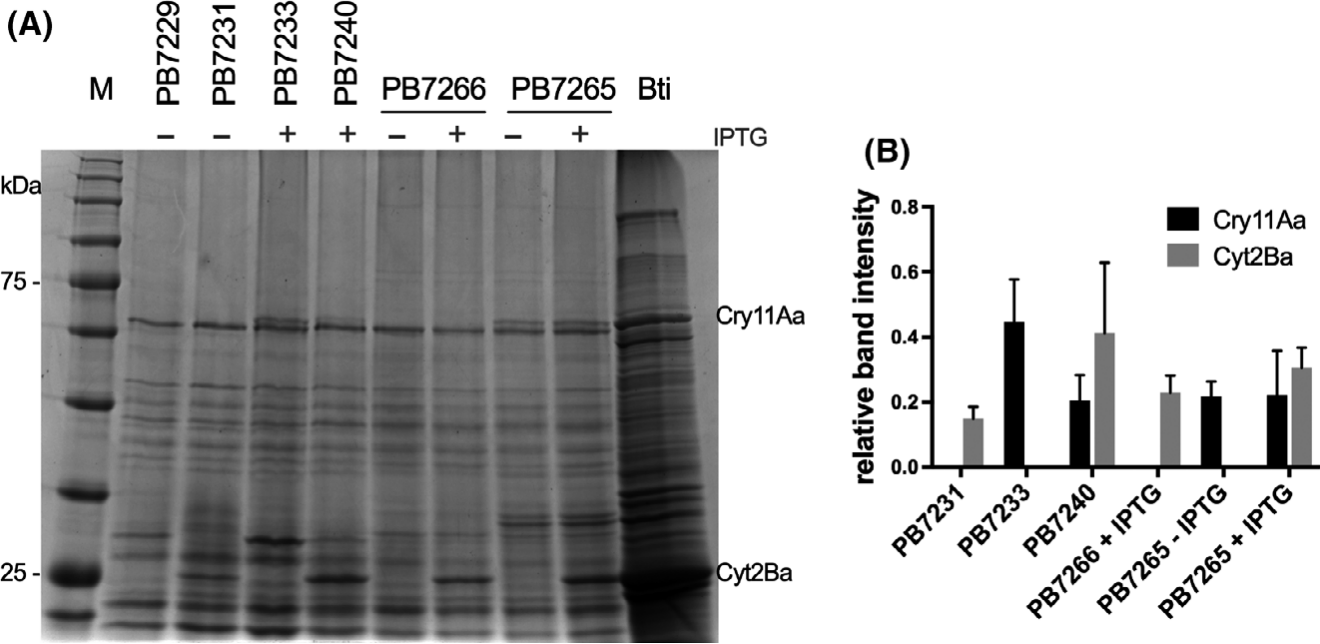
A. 10% SDS‐PAGE analysis of spore–parasporal body mixtures of strains PB7229 (pBS19), PB7231 (pBS19‐*Pcyt2Ba*), PB7233 (*amyE::Phyperspank‐cry11p20*, pBS19 – induced with IPTG 1 mM at T0), PB7240 (*amyE::Phyperspank*‐*cry11p20*, pBS19‐P*cyt2Ba* – induced with IPTG 1 mM at T0), PB7266 (*amyE::Phyperspank‐cyt2Ba)* not induced and induced at T0, PB7265 (*amyE::Phyperspank*‐*cyt2Ba*, *ΔaprE*::*PaprE‐cry11Aa‐p20*) not induced and induced at T0. All strains were grown simultaneously in 2xSG, and the cultures containing cells, spores and parasporal body mixtures were collected 48 h after the beginning of the stationary phase (T48). Bti 4Q1 spore–parasporal bodies collected at T48 were used as positive control. M: (A), Protein Marker VI (10–245) prestained (PanReac). B. Graphical representation of relative Cry11Aa and Cyt2Ba band intensities. Each value represents the average ± SD of three independent replicas. The intensities of the bands corresponding to Cry11Aa and Cyt2Ba were normalized relative to the intensities of the 70 and 22 kDa bands of the protein marker respectively. Each value represents the average ± SD of the three independent replicas.

**Fig. 3:**
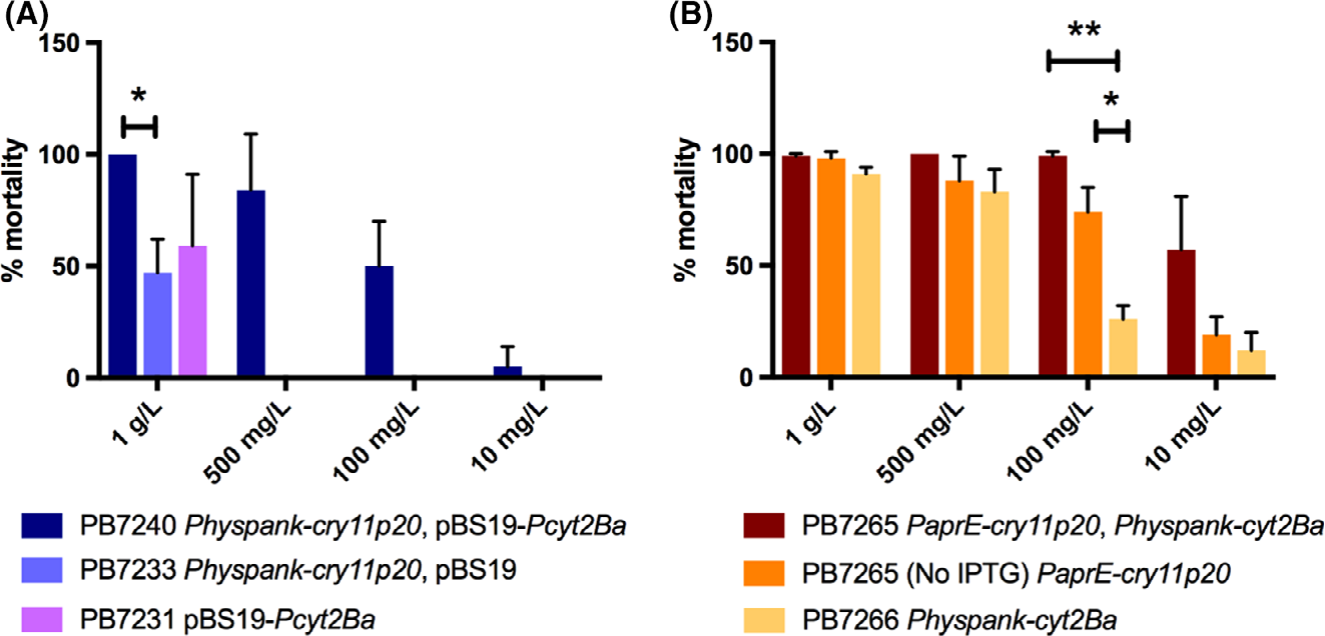
Percentage of *Aedes albopictus* larval mortality at 48 h after the beginning of the assay. The effect of treatment with decreasing concentrations of cells–spores–parasporal bodies mixtures (biomass wet weight l^−1^) collected 48 h after the beginning of the stationary phase was assessed. A. Strains PB7233, PB7231 and PB7240. Statistically significant differences were observed between PB7240 and strains PB7233 (1 g l^−1^, *P = *0.014; 500 mg l^−1^, *P = *0.014; 100 mg l^−1^, *P = *0.024) and PB7231 (500 mg l^−1^, *P = *0.017, 100 mg l^−1^, *P = *0.036). B. Strains PB7265 (not induced and induced with 1 mM IPTG) and PB7266 induced (*amyE::Physpanc‐cyt2Ba*). **P = 0.0002; *P = 0.016. Results are means ± SD of at least three independent experiments, each performed in duplicate.

Strains PB7240 and PB7265 were both able to produce the two Bti toxins but differed in the relative abundance of the two proteins (Fig. 2). In particular, as revealed by quantification of band intensity, coexpression of Cry11Aa and Cyt2Ba in PB7240 resulted in an increase in Cyt2Ba levels and in a decrease in Cry11Aa abundance compared to strains PB7231 and PB7233, expressing the two toxins individually. On the contrary, Cry11Aa and Cyt2Ba protein levels in PB7265 were similar to those observed when the two toxins were expressed individually. While the two strains coexpressing both toxins showed similar levels of Cry11Aa, PB7240 displayed a higher abundance of the cytolytic protein. When assayed for their toxicity against second‐instar larvae of *Ae. albopictus,* PB7240 and PB7265 revealed differences in their larvicidal activities (Fig. 3). Pellets collected 48 h after the beginning of the stationary phase and containing a mix of spore–cells and parasporal bodies were resuspended in dH_2_O and used at decreasing concentrations (expressed as biomass wet weight l^−1^) in larvicidal assays. As assessed at 48 h after the beginning of the assay, after induction, strain PB7265 was more toxic than PB7240, with a statistically significant difference displayed at the assayed concentrations of 100 and 10 mg l^−1^ (*P = *0.013 and 0.025 respectively). Notably, PB7265 was the most toxic engineered *B. subtilis* strain built in this work.

The larvicidal activity of the strains coexpressing Cry11Aa and Cyt2Ba was compared to the one of their corresponding parental strains expressing each toxin individually. As reported in Fig. 3A, when tested at the final concentration of 500 mg l^−1^ or lower, strain PB7240, expressing both toxins, induced a percentage of larval mortality (84% at 500 mg l^−1^; 50% at 100 mg l^−1^; 5% at 10 mg l^−1^) that is higher than the sum of the mortality induced by the strains expressing Cyt2Ba (PB7231) or Cry11Aa (PB7233) alone (0.07% and 0.03% at 500 mg l^−1^, respectively; no mortality induced by any of the two strains at lower concentrations; Fig. 3A). On the contrary, in the *degU32(hy)* strain PB7265, the concomitant expression of Cyt2Ba (IPTG‐induced from a single copy gene) and Cry11Aa (*PaprE‐cry11Aa‐p20*) resulted in a larvicidal activity similar to the sum of the activities of the two toxins expressed individually. This additive effect of the two toxins on larval mortality could be easily appreciated at the assayed concentration of 100 mg l^−1^ (Fig. 3B). At the two highest concentrations (1 g l^−1^ and 500 mg l^−1^) used in the assay, larval mortality reached almost 100% in all tested conditions, masking any significant difference in larvicidal activity between conditions in which the two toxins were expressed individually or in combination.

Worthy of note, strain PB7266, expressing *cyt2Ba* under the control of the P*hyperspank* promoter in a *degUhy* background, shows a significantly higher larvicidal activity when compared to PB7231 (*P = *0.0097, 1 g l^−1^, 48‐h from the beginning of the assay): while the toxicity of PB7266 is detectable even at lower concentrations (up to 10 mg l^−1^), the larvicidal activity of PB7231 can be observed only at the concentration of 1 g l^−1^ (Fig. 3A and B).

## Discussion

In this work, *B. subtilis* was used as a host for the expression of heterologous mosquitocidal toxins. By using diverse expression systems, we created different engineered strains expressing Bti δ‐endotoxins and showing different degrees of larvicidal activity. The toxic activity of three Bti toxins, Cry11Aa, Cyt1Aa and Cyt2Ba, was studied individually or in combination, in different genetic backgrounds and under the control of different promoters.

The ability of recombinant *B. subtilis* to synthesize active δ‐endotoxins of the phylogenetically related bacterium *B. thuringiensis israelensis* was previously demonstrated using *Ae. albopictus* cells (Ward *et al*., 1986; Calogero *et al*., 1989). In this work, for the first time, *Ae. albopictus* larvae were used to test the larvicidal activity of recombinant *B. subtilis* strains expressing Bti δ‐endotoxins.

The ability of *B. subtilis* to express active Bti Cry11Aa endotoxin, with and without the helper protein P20, was evaluated by using an IPTG‐inducible system. Previous studies showed that coexpression of Cry11Aa and P20 in recombinant *B. thuringiensis* (Wu and Federici, 1995) and in Gram‐negative bacteria (Xu *et al*., 2001) resulted in a greater amount of Cry11Aa protein levels and in a higher toxicity against third‐instar *Ae. aegypti* larvae with respect to expression of Cry11Aa protein alone. These previous works were performed by either cloning the two adjacent genes in multicopy plasmids, downstream of their own promoter (Wu and Federici, 1995) or by using the inducible T5 promoter (Xu *et al*., 2001). In this work, the *amyE* integrated *Phyperspank‐cry11Aa* construct displayed a higher efficiency in Cry11Aa expression compared to *Phyperspank‐cry11Aa‐p20* (Fig. S1). A direct correlation between Cry11Aa expression level and the larvicidal activity of the engineered strain was observed (Fig. 1A). The collected results therefore suggest that in *B. subtilis* Cry11Aa can be efficiently expressed even in the absence of P20 and that its expression is sufficient to confer larvicidal activity. We cannot exclude that the unexpected lower expression of Cry11Aa in PB7226 is due to a lower stability of the longer *Phyperspank‐cry11Aa‐p20* transcript compared to *Phyperspank‐cry11Aa*. The lack of a band corresponding to P20 as verified by SDS‐PAGE is consistent with results reported in previous studies (e.g. Wu and Federici, 1995; Tang *et al*., 2006; Valtierra‐de‐Luis *et al*., 2020). The role of P20 in affecting Cry11Aa levels and stability in *B. subtilis* should be further investigated by using combinations of construct expressing *cry11Aa* and *p20* individually.

As the 20‐kDa helper protein was previously reported to be required for efficient expression of Cyt1Aa (Wu and Federici, 1993; Sazhenskiy *et al*., 2010) and other Cry toxins (Shao *et al*., 2001; Diaz‐Mendoza *et al*., 2012; Elleuch *et al*., 2015), the *cry11Aa‐p20* construct was retained for further experiments.

Inducible systems require constant cell‐culture monitoring and their use increases the cost of the commercial product. The generation of a more physiological, auto‐inducible expression system could therefore be useful to overcome the limits associated with IPTG induction. To this purpose, *cry11Aa* and *p20* were cloned under the control of the endogenous *aprE* promoter in *B. subtilis*. In the wild‐type strain, the transcription from the *PaprE* was not strong enough to drive *cry11Aa* expression. Therefore, a *degU32(hy)* mutant strain of *B. subtilis* was used as a host, allowing hyper‐transcription from *PaprE* (Kunst *et al*., 1974; Henner *et al*., 1988; Ogura *et al*., 2004). The auto‐inducible system drove the expression of the heterologous protein during the stationary phase and concurrently to the onset of sporulation, concomitantly with the activation of *PaprE* (Fig. S2). By affecting only one of the regulators (DegU) controlling the expression of *aprE*, a good level of synthesis of the heterologous protein was achieved, leading also to a good level of toxicity. Worthy of note, the intrinsic instability of integrations by single cross‐over leads to the presence of a variable number of the *PaprE‐cry11Aa‐p20* construct on the chromosome: low concentrations of antibiotic in the medium should ensure an average of 1–2 copies per cell.

Cyt1Aa toxin is a cytolytic protein able to synergize with other Cry toxins such as Cry4A, Cry4Ba and Cry11Aa (Wu and Chang, 1985; Crickmore *et al*., 1995; Poncet *et al*., 1995). It was demonstrated that Cyt1Aa functions as a Cry11Aa membrane receptor (Perez *et al*., 2005). The interaction between the two proteins supports toxicity both by pore formation and by oligomerization of Cry11Aa toxin (Soberon *et al*., 2013). Cyt1Aa expression in *B. subtilis* was previously reported to cause mortality against third‐instar *Ae. aegypti* larvae (Ward *et al*., 1986). However, in this work, when the same *cyt1Aa‐p21* region was cloned downstream of the IPTG‐inducible *Phyperspank* promoter and integrated in the *B. subtilis* chromosome, no detectable levels of Cyt1Aa could be observed. On the contrary, the cytolytic protein was highly produced by cloning the same construct downstream of its own promoter, in a multicopy plasmid (pBS19‐*cyt1Aa‐p21*; Fig. S4). The high relative abundance of Cyt1Aa in the Bti parasporal body can be attributed to different factors, including the level of *cyt1Aa* gene expression, as well as its transcript and protein stability. While both the *Phyperspank‐cyt1Aa‐p21* and the pBS19*‐ cyt1Aa‐p21* constructs include the 3’‐UTR stem‐loop structure involved in *cyt1Aa* transcription termination and mRNA stability (Sakano *et al*., 2017), they differ in their copy number and in the promoter used to drive gene expression. Both these elements were previously reported to affect Cyt1Aa levels. In Bti, the expression of *cyt1Aa* is controlled by three functional non‐overlapping promoters (BtI, BtII and BtIII) that are activated during sporulation by σ^E^ (BtI and BtIII) and σ^K^ (BtII) transcription factors (Sakano *et al*., 2017). While the combination of BtI, BtII and BtIII leads to the highest level of *cyt1Aa* expression, transcript levels can be modulated by using independent or different combinations of Bt promoters. In this work, the 1224 bp region cloned in pBS19 and comprising the *cyt1Aa* gene and the region upstream of its cds include all three Bt promoters for full *cyt1Aa* expression. An additional factor influencing Cyt1Aa protein production is plasmid copy number. At least four copies of a plasmid carrying the *cyt1Aa* gene downstream of the BtIII promoter must be present in Bti to obtain detectable Cyt1Aa levels (Park *et al*., 2016). Interestingly, no synthesis of Cyt1Aa was detected in Bti recombinant strains transformed with a low copy number (2–3) plasmid carrying a pBtoxis minireplicon comprising the *cyt1Aa* gene and its three Bt promoters (Tang *et al*., 2006). We therefore hypothesize that the presence of a single copy of *cyt1Aa* might be the reason of the lack of efficient Cyt1Aa protein production in PB7232. In this strain, the *Phyperspank* promoter is unable to drive sufficient expression of the cytotoxic gene. On the contrary, the pBS19‐*cyt1Aa‐p21* construct, including all three Bt promoters and present in multiple copies per cell, allowed achieving a high level of Cyt1Aa expression. Notably, high Cyt1A expression resulted to be cytotoxic against *B. subtilis* itself (Table S3, Fig. S5). As overexpression of Cyt1Aa toxin in Bti was previously reported to result in significantly fewer spores per unit medium and in the formation of imperfect crystals (Park *et al*., 2016), it can be hypothesized that, even in *B. thuringiensis israelensis*, Cyt1Aa expression needs to be tightly regulated to prevent off‐target toxicity.

Despite different studies described the role of Cyt1Aa in enhancing the toxicity of Bti and in delaying the insurgence of resistance, the interactions of Cyt2Ba with other components of the Bti crystals are still poorly investigated. Synergy between Cyt2Ba and Cry4Aa proteins was previously reported against larvae of *Ae. aegypti* (Manasherob *et al*., 2006). Moreover, very recently, the co‐administration of two recombinant Bt strains, each expressing Cry10 or Cyt2Ba, respectively, revealed a synergistic interaction between the two toxins when simultaneously ingested by *A. aegypti* larvae (Valtierra‐de‐Luis *et al*., 2020). Here, the possible interaction between Cry11Aa and Cyt2Ba was investigated.

We constructed mosquitocidal *B. subtilis* strains in which *cyt2Ba* was cloned either in single copy, downstream of the *amyE* integrated *Phyperspank* promoter, or in the multicopy vector pBS19, under the control of its own promoter. The cytolytic toxin was expressed efficiently in both constructs, conferred larvicidal activity and its expression was not lethal for the host. We therefore decided to study the possible synergism between Cyt2Ba and Cry11Aa. To this purpose, strains PB7240 (*Phyperspank‐cry11Aa‐p20* pBS19‐*Pcyt2Ba*) and PB7265 (*PaprE‐cry11Aa‐p20, amyE::Phyperspank*‐*cyt2Ba*) were prepared.

In strain PB7240, the coexpression of the two toxin genes resulted in a twofold increase in Cyt2Ba levels and in a concomitant twofold decrease in Cry11Aa abundance with respect to the corresponding parental strains (PB7231 and PB7233) expressing each toxin individually. As the protein P20 was previously reported to be involved in the stabilization of Cyt1Aa (Visick and Whiteley, 1991; Wu and Federici, 1993), we may hypothesize that it may also contribute to a higher stability of Cyt2Ba. Similarly to what described in *E. coli* for Cyt1Aa, P20 may prevent Cyt2Ba proteolytic degradation, leading to higher levels of the cytolytic protein in strain PB7240 compared to PB7231. The increase in Cyt2Ba levels in PB7240 was concomitant to a decrease in Cry11Aa yields with respect to PB7233, possibly due to energy expenditure dedicated the synthesis of the cytolytic protein. Strain PB7240 displayed a larvicidal activity higher than the sum of larval mortality induced by the corresponding strains expressing each toxin individually (Fig. 3A). This increase in larvicidal activity cannot be ascribed only to the twofold increase in Cyt2Ba levels, since at concentrations at which no larvicidal activity can be observed for strain PB7231 (e.g. at 100 mg l^−1^), PB7240 can still induce a larval mortality similar to the one induced by PB7231 when used at a 10‐fold higher concentration. These observations suggest a synergism between Cry11Aa and Cyt2Ba in this genetic background.

On the contrary, an additive effect between the two toxins was observed when using a *degU32(hy)* and protease‐deficient (Δ*aprE* and Δ*nprE*) *B. subtilis* strain. In this genetic background, the IPTG‐dependent expression of *cyt2BA* in PB7266 allowed obtaining levels of cytolytic protein similar to those displayed by PB7231. However, in contrast to what observed in PB7240, no increase in Cyt2Ba levels was observed in PB7265 when both toxins were produced: after IPTG induction, PB7265 displayed levels of Cyt2Ba similar to those observed in strain PB7266 (+ IPTG). As detected in the parental strain PB7241 (Fig. S2), being the P*aprE* promoter activated at the beginning of sporulation, its use for driving gene expression leads to the accumulation of the produced proteins during late stationary phase. On the contrary, in PB7233, expression of the *Phyperspank‐cry11Aa‐p20* construct is induced earlier, i.e. at the moment of transition from exponential to stationary phase. The delayed expression of *cry11Aa* in PB7265 compared to PB7233 results in a lower level of Cry11Aa toxin observed 48 h after the beginning of the stationary phase. For the same reason, the amount of P20 produced by PB7265 could be insufficient to exert any effect on the stability of Cy2Ba, whose levels remain similar in PB7266 and PB7265. Notably, in the absence of an increase in Cyt2Ba, the ability of PB7265 to produce Cry11Aa is not affected by the IPTG‐dependent induction of P*hyperspank‐cyt2Ba*.

It is noteworthy that, despite expressing similar or even lower levels of Cry11Aa and Cyt2Ba, PB7266 and PB7265 displayed a significantly higher larvicidal activity compared to PB7231, PB7233 and PB7240. We may speculate that the lack of the two major *B. subtilis* exoproteases may allow a higher stability of the produced proteins, resulting in a higher toxicity of the strains expressing the two toxins, individually or in combination.

The results here obtained demonstrate that *B. subtilis* can be efficiently used as a host system to study the activity of individual or combinations of toxins identified in mosquitocidal bacterial strains. The deep knowledge of its genetics, together with the availability of well‐established tools for its manipulation, allows the modulation and optimization of heterologous toxin production. The expression systems here presented lay the foundation for the construction of a better improved system that might be used to characterize, modulate and evaluate the level of expression and the larvicidal activity of individual toxin genes from new environmental isolates.

## Experimental procedures

### Bacterial strains, plasmids, primers and growth conditions

The *Bacillus thuringiensis israelensis* strain 4Q1 (Bacillus Genetic Stock Center, original code HD567 (Goldberg and Margalit, 1977)) was used as source of template DNA for cloning and as positive control in δ‐endotoxins expression analysis and in larvicidal assays. The *B. subtilis* bacterial strains constructed and used in this work are listed in Table 1 and prepared as described in Supplementary Information. *Escherichia coli* DH5α was used for construction and maintenance of plasmids (Table S2). The primers and plasmids used in this work are listed in Tables S1 and S2 respectively. Growth media employed in this work include Luria‐Bertani medium (LB), LM, MD and MDCH (Kunst and Rapoport, 1995) and 2xSG sporulation medium: 16 g l^−1^ of Nutrient Broth (Difco), 2 g l^−1^ KCl, 0.5 g l^−1^ MgSO_4_*7H_2_O, 1 mM Ca(NO_3_)_2_, 0.1 mM MnCl_2_*4H_2_O, 1 M FeSO_4_ and 0.1% glucose (Leighton and Doi, 1971). Antibiotics were used at the following concentrations: ampicillin, Amp (for *E. coli*) 100 µg ml^−1^; chloramphenicol, Caf 5 µg ml^−1^; spectinomycin, Spc 100 µg ml^−1^; kanamycin, kana 20 µg ml^−1^, erythromycin, Ery 0.5 µg ml^−1^; and lincomycin, Linc 12.5 µg ml^−1^.

**Table 1:** *Bacillus subtilis* strains used in this study.

Strain name	Genotype	Source or origin
PB168	*trpC2*	BGSC^a^ strain 1A1
PB1831	*trpC2, phe‐1*	BGSC^a^ strain 1A96 (JH642, J. Hoch)
PB7007	*ΔaprE, ΔnprE, thr‐, amyE::[comK (ery)], degU32H*	University of Pavia Collection
PB7222	*trpC2, phe‐1, amyE::spc*	PB1831 × pDR111
PB7223	*trpC2, phe‐1, amyE::Physpank‐cry11Aa*	PB1831 × pDR111‐*cry11Aa*
PB7225	*trpC2, phe‐1, amyE::Physpank‐cyt2Ba*	PB1831 × pDR111‐*cyt2Ba*
PB7226	*trpC2, phe‐1, amyE::Physpank‐cry11Aa‐p20*	PB1831 × pDR111‐*cry11Aa‐p20*
PB7229	*trpC2, phe‐1, pBS19*	PB1831 × pBS19
PB7230	*trpC2, phe‐1, pBS19‐Pcyt1Aa‐p21*	PB1831 × pBS19‐*Pcyt1Aa‐p21*
PB7231	*trpC2, phe‐1, pBS19‐Pcyt2Ba*	PB1831 × pBS19‐*Pcyt2Ba*
PB7232	*trpC2, phe‐1, amyE::Physpank‐cyt1Aa‐p21*	PB1831 × pDR111‐*cyt1Aa‐p21*
PB7233	*trpC2, phe‐1, amyE::Physpank‐cry11Aa‐p20, pBS19*	PB7226 × pBS19
PB7240	*trpC2, phe‐1, amyE::Physpank‐cry11Aa‐p20, pBS19‐Pcyt2Ba*	PB7226 × pBS19‐*Pcyt2Ba*
PB7241	*ΔnprE, thr‐, amyE::[comK (ery)], degU32H ΔaprE::PaprEcry11Aap20*	PB7007 × pBG105
PB7242	*ΔnprE, thr‐, amyE::[comK (ery)], degU32H ΔaprE::PaprE*	PB7007 × pBG109
PB7246	*ΔaprE, ΔnprE, thr‐, amyE::spc, degU32H*	PB7007x PB7222
PB7247	*ΔaprE, ΔnprE, thr‐, amyE::Physpank‐cry11Aa‐p20, degU32H*	PB7007 × PB7226
PB7265	*ΔnprE, thr‐, amyE::Physpank‐cyt2Ba, degU32H ΔaprE::PaprEcry11Aap20*	PB7241 × PB7225
PB7266	*ΔnprE, thr‐, amyE::Physpank‐cyt2Ba, degU32H ΔaprE::PaprE*	PB7242 × PB7225

^a^ BGSC: *Bacillus* Genetic Stock Center culture collection catalogue number.

### Preparation of spores, parasporal bodies and cells mixtures

Engineered *B. subtilis* strains were cultured in 10 ml LB overnight. The following day, the o/n preculture was diluted to OD_600_ = 0.05 in 20 ml of 2xSG sporulation medium and incubated with shaking to OD_600_ = 1. Then, the culture was re‐diluted to OD_600_ = 0.1 in 60 ml of fresh 2xSG and grown at 37 °C, 250 rpm. Heterologous gene expression driven by the P*hyperspank* promoter was induced with 1 mM IPTG at the beginning of the stationary phase (T0). At the time of collection, 20 ml of culture containing whole spores, cells and parasporal bodies were harvested and then pelleted by centrifugation (10 000 rpm × 15’ 4 °C). The pellets were then suspended in Tris‐HCl 10 mM (pH = 7) and aliquots of 1 ml were prepared. After a new centrifugation at 12 000 *g* × 10’ at 4 °C, the pellets were stored at −20 °C or immediately used for protein analysis by SDS‐PAGE 10%, sporulation and larvicidal assay.

### Protein detection and quantification

Pellets of spores–crystal mixtures were resuspended in dH_2_O to a final concentration of 200 mg ml^−1^ wet weight/volume. Samples (15 µl) were heated in a thermocycler at 95 °C for 5 min in loading buffer (0.05 M Tris pH 6.8, 10% glycerol, 2% SDS, 5% β‐mercaptoethanol and 0.05% bromophenol blue) and subjected to electrophoresis in 10% SDS‐PAGE. Gels were stained with Coomassie Brilliant Blue. Images were acquired using a Bio‐Rad ChemiDoc MP Imaging System. Quantification of bands intensities was performed using ImageJ Software (Schneider *et al*., 2012).

### Larvicidal assay

The larvicidal activity of the engineered *Bacillus subtilis* strains was analysed by mortality assays. Twenty‐five second‐instar larvae of *Aedes albopictus* Rimini strain reared at 28 °C, in a 12 h‐light/12 h‐dark photoperiod, with 70% of humidity, were placed in 100 ml of deionized water and were fed with mixtures of engineered *B. subtilis* spores, parasporal bodies and cells. Mixtures were added at different concentrations, expressed as biomass wet weight/litre. Mortality was recorded after 24, 48 and 72 h from the beginning of the assay. No nutritional supplement was added. The experiments were carried out at room temperature, and each test was conducted with three independent culture replicates. 4Q1 Bti spores, parasporal bodies and cells collected after 72 h of growth in 2xSG were used as positive controls. Not‐induced cultures and cells transformed with empty vectors were used as negative controls.

### Sporulation assay

To calculate the number of heat‐resistant spores, appropriate dilutions of the resuspended pellets were plated on LB plates supplemented with antibiotic (when needed) before and after treatment at 80 °C for 10 min. The percentage of sporulation was calculated according to the following formula:

$$\frac{\text{No of CFUs/ml grown after heat ‐ treatment}}{\text{No of CFUs/ml grown before heat ‐ treatment}}\times 100$$

### Statistical analysis

Statistical analysis was performed on ≥ 3 independent experiments using GraphPad Prism version 8 software. Statistical significance was determined using the Holm–Sidak method, with alpha = 0.05.

## Conflict of interest

None declared.

## Supporting information


**Fig. S1** Expression of the *cry11Aa* Bti toxin gene in *B. subtilis* under the control of the *Phyperspank* promoter. A. Schematic representation of the *Phyperspank‐cry11Aa* and *Phyperspank‐cry11Aa‐p20* constructs integrated by double cross‐over in the *amyE* gene of *B. subtilis* PB1831. B. Growth of strains PB7222 (*amyE::spc*), PB7223 (*amyE::Phyperspank‐cry11Aa*) and PB7226 (*amyE::Phyperspank‐cry11Aa‐p20*) in 2xSG sporulation medium. Heterologous protein expression was induced with 1 mM IPTG at T0, defined as the time point of transition from exponential to stationary phase of growth. C. SDS‐PAGE 10% of cells‐spores‐parasporal bodies mixtures (15 μl of 200 mg/ml [wet weight/vol] suspension/well) of strains PB7222, PB7223 and PB7226 collected 20 hours after the beginning of the stationary phase (T20). Bti: 4Q1 *B. thuringiensis* *israelensis* spore‐parasporal bodies mixtures collected at T72 as positive control. M: PageRuler Unstained Protein Ladder. Strain PB7226 displays a 15‐fold lower Cry11Aa protein level relative to PB7223, as quantified by band intensity using ImageJ software. Band intensity was normalized with respect to the 70 kDa band of the protein marker.

**Fig. S2** Expression of the *cry11Aa* toxin gene in *B. subtilis* under the control of the *PaprE* promoter. A.Schematic representation of the *PaprE‐cry11Aa‐p20* construct integrated by single cross‐over in the regulatory region of the *aprE* gene in *B. subtilis* PB1831. B. Growth of PB7241 (*PaprE‐cry11Aa‐p20*) and PB7242 (*PaprE*) in 2xSG medium. Data are the average ± SD of two independent experiments. C. SDS‐PAGE 10% of PB7241 cells‐spore‐parasporal bodies collected at different time points (4, 15, 20 24, 48, 72 hours) after the beginning of the stationary phase (a time point indicated as T0). Bti: Spore‐parasporal bodies of *B. thuringiensis *israelensis 4Q1 collected at T72. Fifteen μl of cells‐spores‐parasporal bodies suspensions at the concentration of 200 mg/ml [wet weight/vol] were loaded in each well. M: PageRuler Unstained Protein Ladder.

**Fig. S3** A. Growth of the *degU32(hy)* strains PB7241 (*PaprE‐cry11Aa‐p20*) and PB7247 (*amyE::Phyperspank‐cry11Aa‐p20*) in 2xSG medium. Data are the average ±SD of two independent experiments. B. SDS‐PAGE 10% of cells‐spores‐parasporal bodies of strains PB7241, PB7247 and of the respective control strains PB7242 (*aprE::PaprE‐kanR, degU32(hy)*) and PB7246 (*amyE::spc, degU32(hy)* collected after 48 hours from the beginning of the stationary phase. The strains PB7246 and PB7247 were IPTG induced at T0. Intensity of the Cry11Aa band of the two strains was quantified using ImageJ software and normalized relative to the intensity of the 63 kDa band of the protein marker. Bti: 4Q1 spore‐parasporal bodies collected at T48. M: Protein Marker VI (20‐345) prestained (PanReac).

**Fig. S4** SDS‐PAGE 10% of the spore‐parasporal body mixtures of the strains PB7222 (*amyE::spc*), PB7232 (*amyE::Phyperspank‐cyt1Aa‐p21*), PB7230 (pBS19‐*Pcyt1Aa‐p21*) and PB7229 (pBS19) collected after 20 hours from the beginning of the stationary phase. PB7222 and PB7232 strains were IPTG induced at T0. Bti 4Q1 spore‐parasporal bodies collected at T72 was used as positive control; M: PageRuler Unstained Protein Ladder.

**Fig. S5** TEM (7000x) of PB7230 (pBS19‐*Pcyt1Aa‐p21*) strain collected at 72 hours from the beginning of the stationary phase. Expression of Cyt1Aa causes death of *B. subtilis* cells.

**Fig. S6** A. Growth of *B. subtilis* recombinant strains expressing Cyt2Ba. Strains PB7222 (*amyE::spc*), PB7225 (*amyE::Phyperspank‐cyt2Ba*), PB7229 (pBS19) and PB7231 (pBS19‐*Pcyt2Ba*) were grown in 2xSG medium. Data are the average ± SD of two independent experiments. B. SDS‐PAGE 10% of the cells‐spores‐parasporal body mixtures of the strains PB7222 (*amyE::spc*), PB7225 (*amyE::Phyperspank‐cyt2Ba*), PB7229 (pBS19) and PB7231 (pBS19‐*Pcyt2Ba*). PB7222 and PB7225 were IPTG induced at T0. All the strains were collected 24 hours after the beginning of the stationary phase. Bti: 4Q1 spore‐parasporal bodies collected at T72. M: PageRuler Unstained Protein Ladder. Intensity of the Cyt2Ba band of strains PB7231 and PB7225 was quantified using ImageJ software and normalized relative to the intensity of the 25 kDa band of the protein marker.

**Fig. S7** Growth of *B. subtilis* recombinant strains expressing Cyt2Ba (PB7231, PB7266 + IPTG), Cry11Aa (PB7233, PB7265) or both toxins (PB7240 and PB7265 + IPTG) compared to control strains PB7229 and PB7266. All strains were grown in 2xSG medium. Data from a single representative experiments are reported.

**Table S1.** Primers used for cloning. Restriction sites are shown in capital letters.

**Table S2.** Plasmids used in this work.

**Table S3.** Titer of cells and spores (cfu ml^−1^) in spore‐parasporal bodies mixtures resuspended at the concentration of 100 mg l^−1^. Results refer to the following strains: PB7229 (negative control, pBS19) and PB7230 (pBS19‐*Pcyt1Aa‐p21*), collected at 24, 48, 72 h after the beginning of the stationary phase (T24, T48, T72). Results are means of three replicas ± SD.

**Supplementary Information.** Genetic techniques employed in the study and detailed description of the preparation of the *B. subtilis* strains expressing δ‐endotoxins.
