# Supplementary figures and images for: Bacillus subtilis as a host for mosquitocidal toxins production
Source: Microb Biotechnol. 2020 Aug 30;13(6):1972–82. doi: 10.1111/1751-7915.13648 (PMC7533320; doi:10.1111/1751-7915.13648)

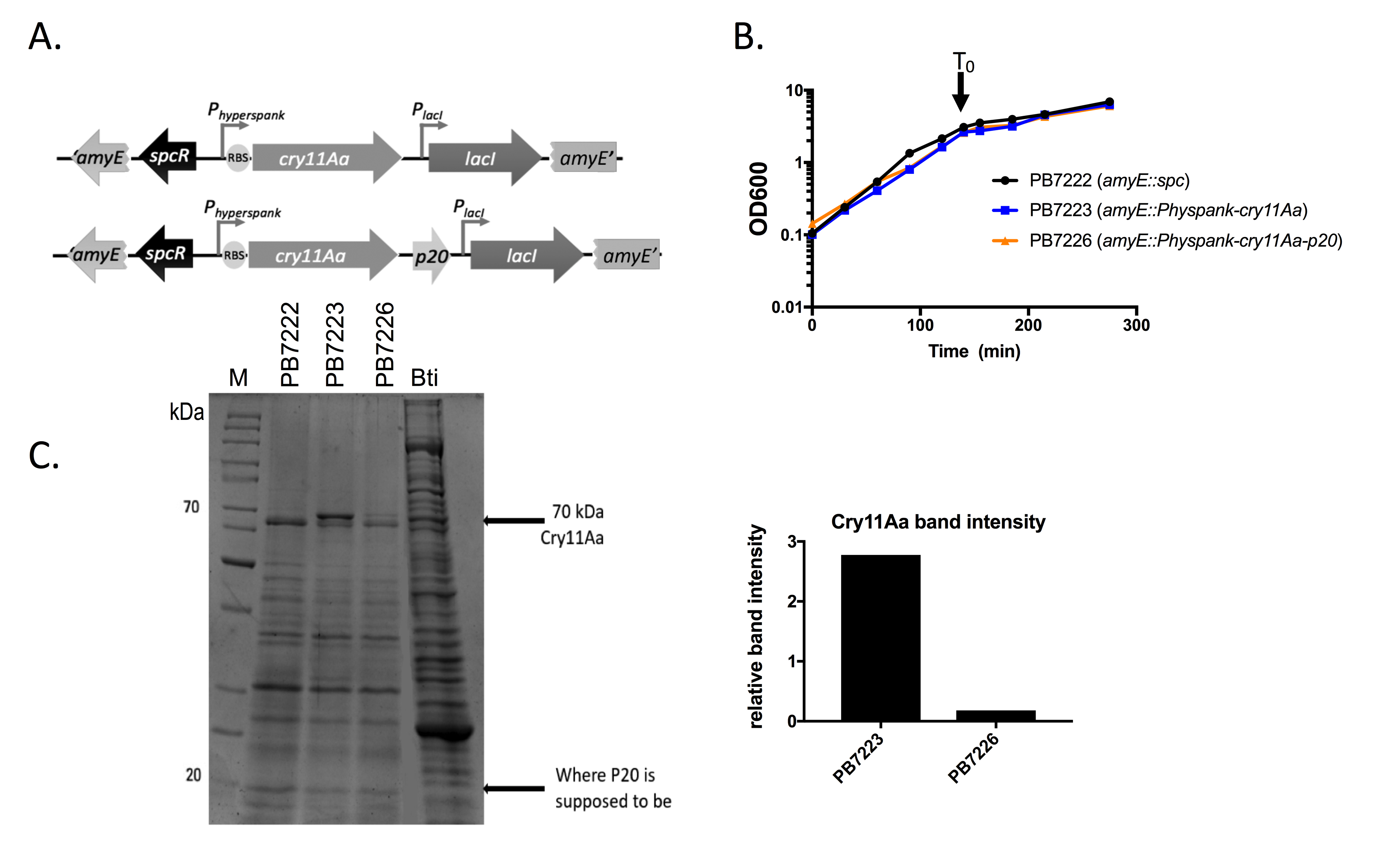

Supplement: Supplementary file 1 — Fig. S1 Expression of the cry11Aa Bti toxin gene in B. subtilis under the control of the Phyperspank promoter. A. Schematic representation of the Phyperspank‐cry11Aa and Phyperspank‐cry11Aa‐p20 constructs integrated by double cross‐over in the amyE gene of B. subtilis PB1831. B. Growth of strains PB7222 (amyE::spc), PB7223 (amyE::Phyperspank‐cry11Aa) and PB7226 (amyE::Phyperspank‐cry11Aa‐p20) in 2xSG sporulation medium. Heterologous protein expression was induced with 1 mM IPTG at T0, defined as the time point of transition from exponential to stationary phase of growth. C. SDS‐PAGE 10% of cells‐spores‐parasporal bodies mixtures (15 μl of 200 mg/ml [wet weight/vol] suspension/well) of strains PB7222, PB7223 and PB7226 collected 20 hours after the beginning of the stationary phase (T20). Bti: 4Q1 B. thuringiensis israelensis spore‐parasporal bodies mixtures collected at T72 as positive control. M: PageRuler Unstained Protein Ladder. Strain PB7226 displays a 15‐fold lower Cry11Aa protein level relative to PB7223, as quantified by band intensity using ImageJ software. Band intensity was normalized with respect to the 70 kDa band of the protein marker. [file MBT2-13-1972-s001.tiff]

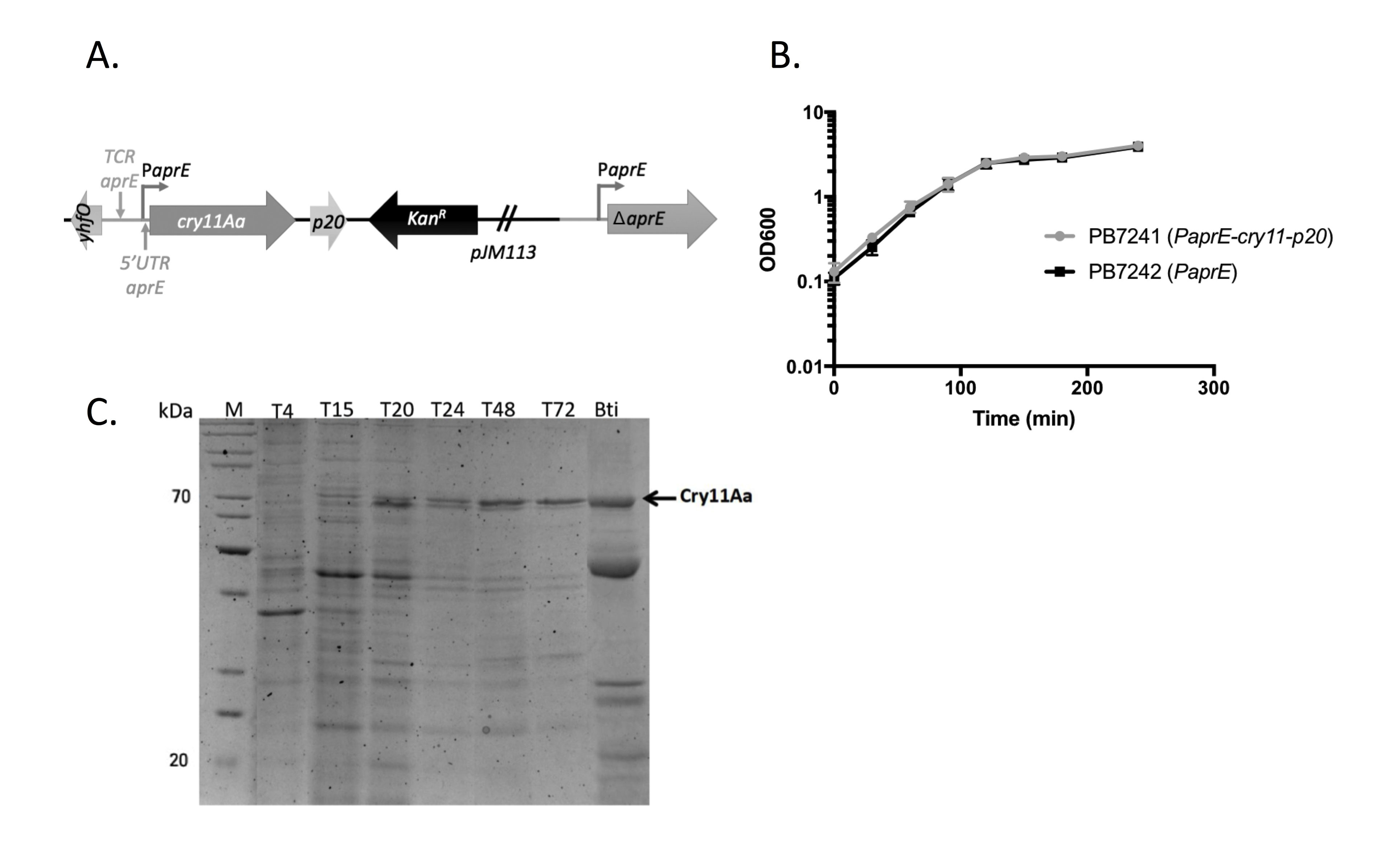

Supplement: Supplementary file 2 — Fig. S2 Expression of the cry11Aa toxin gene in B. subtilis under the control of the PaprE promoter. A.Schematic representation of the PaprE‐cry11Aa‐p20 construct integrated by single cross‐over in the regulatory region of the aprE gene in B. subtilis PB1831. B. Growth of PB7241 (PaprE‐cry11Aa‐p20) and PB7242 (PaprE) in 2xSG medium. Data are the average ± SD of two independent experiments. C. SDS‐PAGE 10% of PB7241 cells‐spore‐parasporal bodies collected at different time points (4, 15, 20 24, 48, 72 hours) after the beginning of the stationary phase (a time point indicated as T0). Bti: Spore‐parasporal bodies of B. thuringiensis israelensis 4Q1 collected at T72. Fifteen μl of cells‐spores‐parasporal bodies suspensions at the concentration of 200 mg/ml [wet weight/vol] were loaded in each well. M: PageRuler Unstained Protein Ladder. [file MBT2-13-1972-s002.tiff]

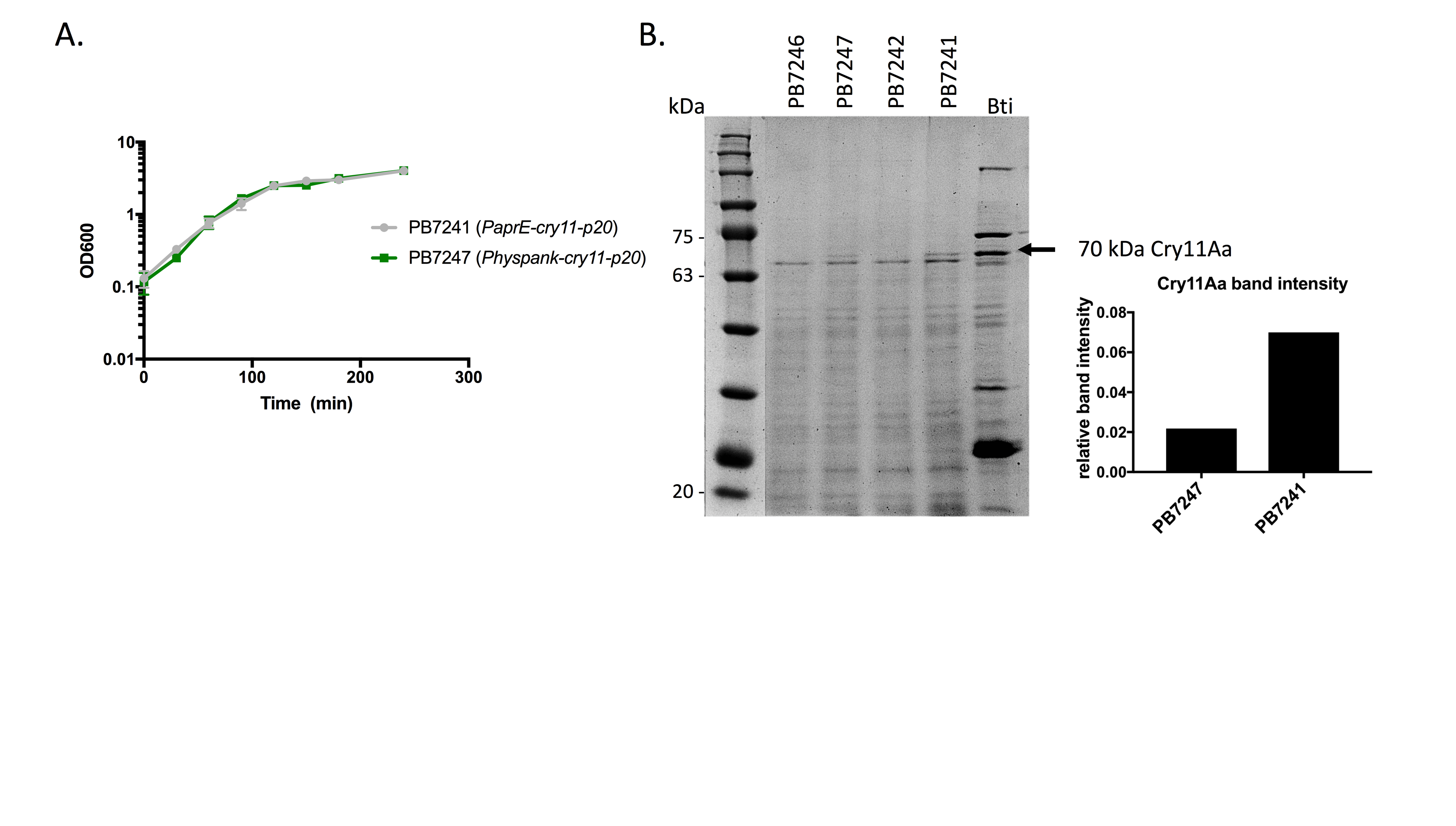

Supplement: Supplementary file 3 — Fig. S3 A. Growth of the degU32(hy) strains PB7241 (PaprE‐cry11Aa‐p20) and PB7247 (amyE::Phyperspank‐cry11Aa‐p20) in 2xSG medium. Data are the average ±SD of two independent experiments. B. SDS‐PAGE 10% of cells‐spores‐parasporal bodies of strains PB7241, PB7247 and of the respective control strains PB7242 (aprE::PaprE‐kanR, degU32(hy)) and PB7246 (amyE::spc, degU32(hy) collected after 48 hours from the beginning of the stationary phase. The strains PB7246 and PB7247 were IPTG induced at T0. Intensity of the Cry11Aa band of the two strains was quantified using ImageJ software and normalized relative to the intensity of the 63 kDa band of the protein marker. Bti: 4Q1 spore‐parasporal bodies collected at T48. M: Protein Marker VI (20‐345) prestained (PanReac). [file MBT2-13-1972-s003.tiff]

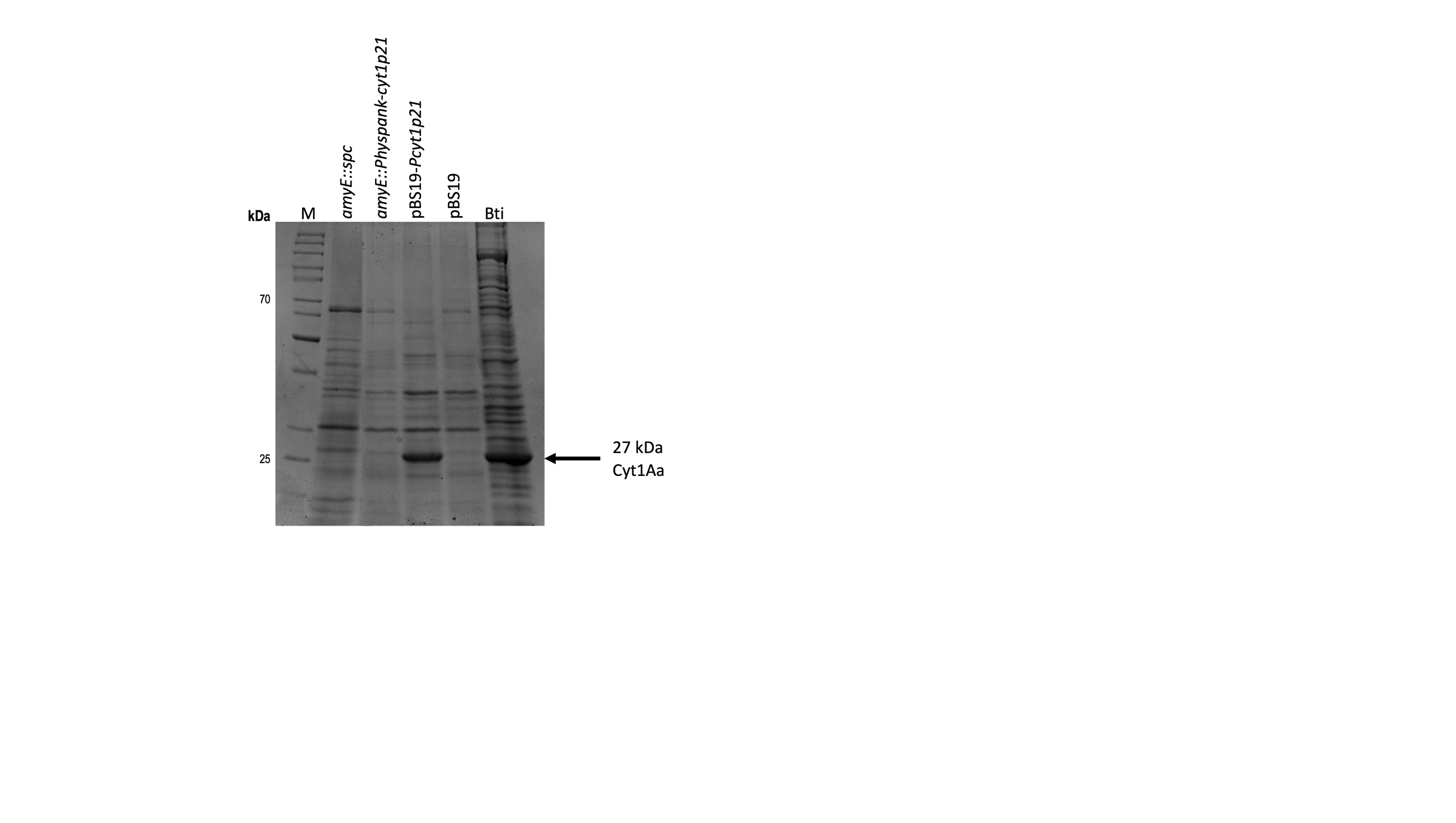

Supplement: Supplementary file 4 — Fig. S4 SDS‐PAGE 10% of the spore‐parasporal body mixtures of the strains PB7222 (amyE::spc), PB7232 (amyE::Phyperspank‐cyt1Aa‐p21), PB7230 (pBS19‐Pcyt1Aa‐p21) and PB7229 (pBS19) collected after 20 hours from the beginning of the stationary phase. PB7222 and PB7232 strains were IPTG induced at T0. Bti 4Q1 spore‐parasporal bodies collected at T72 was used as positive control; M: PageRuler Unstained Protein Ladder. [file MBT2-13-1972-s004.tiff]

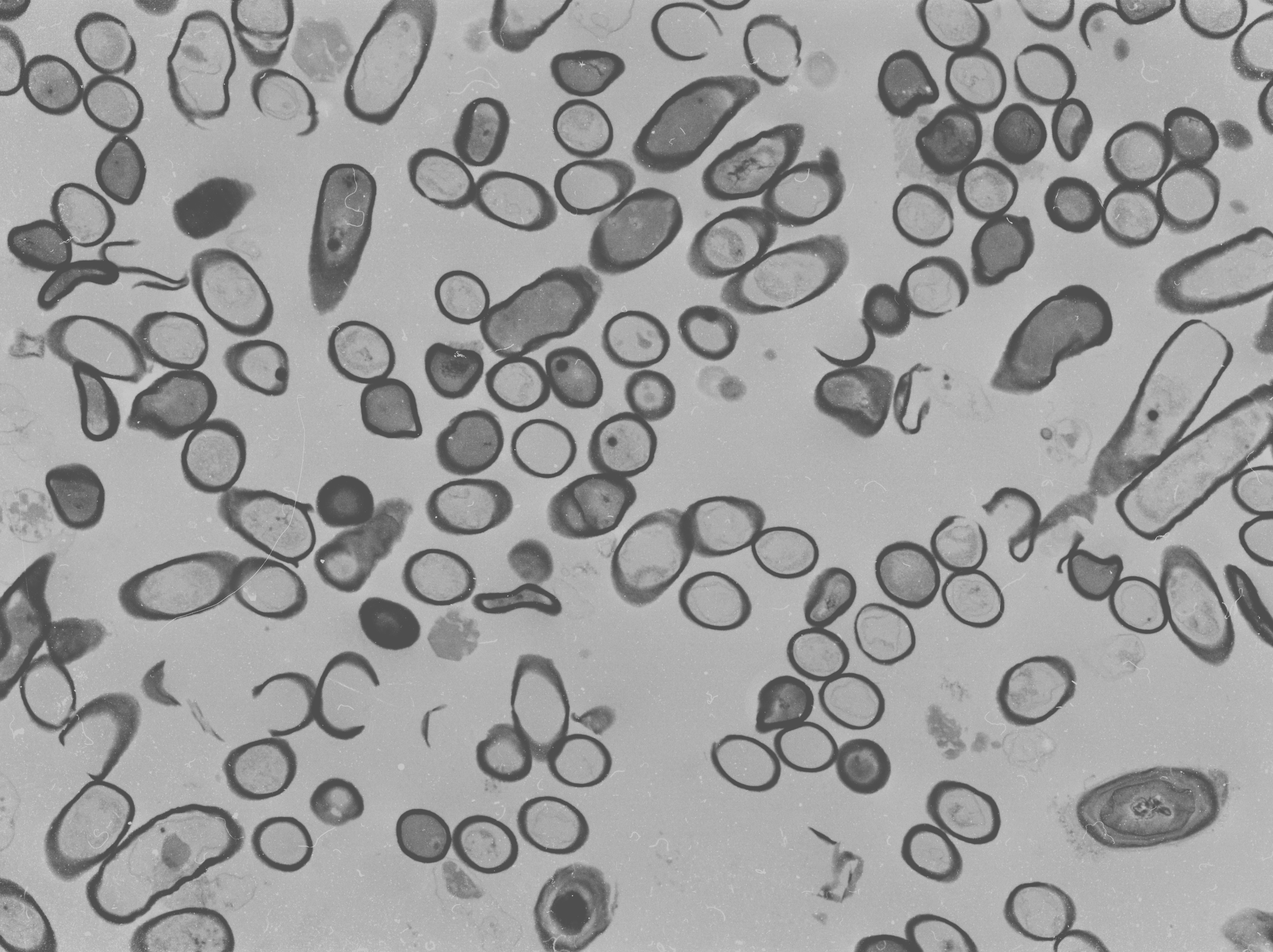

Supplement: Supplementary file 5 — Fig. S5 TEM (7000x) of PB7230 (pBS19‐Pcyt1Aa‐p21) strain collected at 72 hours from the beginning of the stationary phase. Expression of Cyt1Aa causes death of B. subtilis cells. [file MBT2-13-1972-s005.tif]

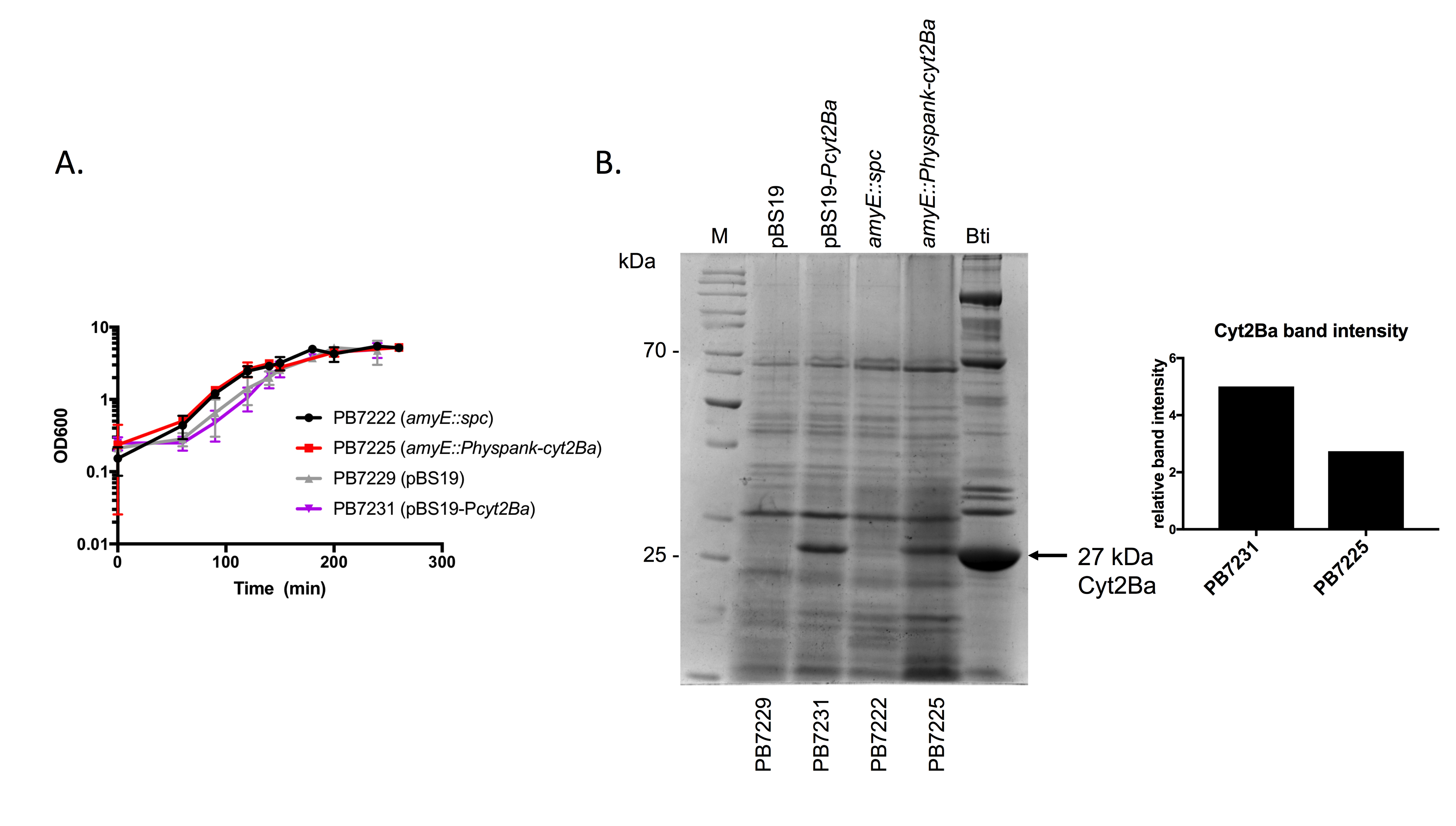

Supplement: Supplementary file 6 — Fig. S6 A. Growth of B. subtilis recombinant strains expressing Cyt2Ba. Strains PB7222 (amyE::spc), PB7225 (amyE::Phyperspank‐cyt2Ba), PB7229 (pBS19) and PB7231 (pBS19‐Pcyt2Ba) were grown in 2xSG medium. Data are the average ± SD of two independent experiments. B. SDS‐PAGE 10% of the cells‐spores‐parasporal body mixtures of the strains PB7222 (amyE::spc), PB7225 (amyE::Phyperspank‐cyt2Ba), PB7229 (pBS19) and PB7231 (pBS19‐Pcyt2Ba). PB7222 and PB7225 were IPTG induced at T0. All the strains were collected 24 hours after the beginning of the stationary phase. Bti: 4Q1 spore‐parasporal bodies collected at T72. M: PageRuler Unstained Protein Ladder. Intensity of the Cyt2Ba band of strains PB7231 and PB7225 was quantified using ImageJ software and normalized relative to the intensity of the 25 kDa band of the protein marker. [file MBT2-13-1972-s006.tiff]

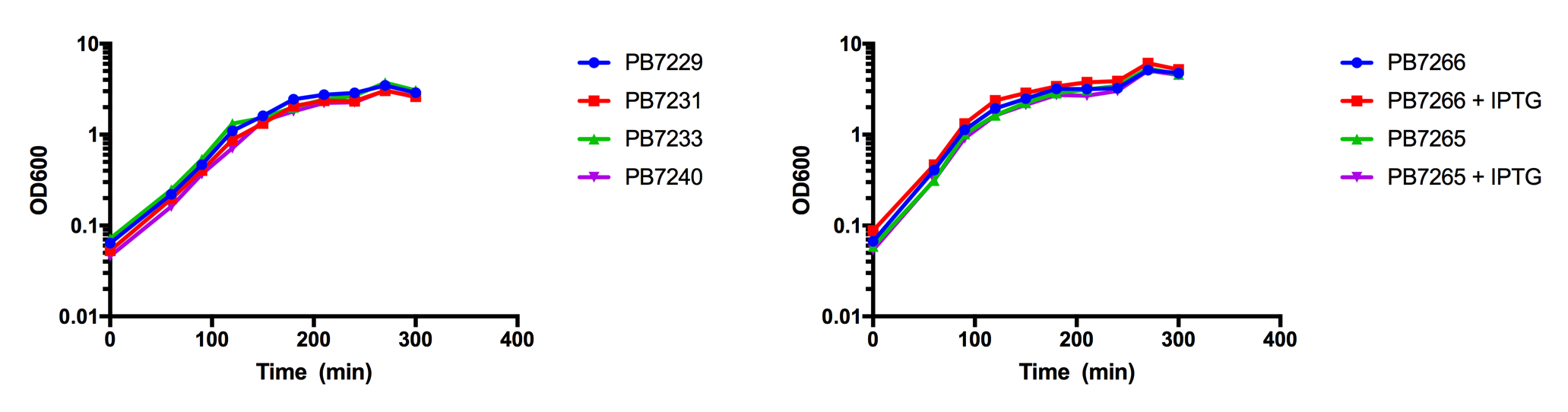

Supplement: Supplementary file 7 — Fig. S7 Growth of B. subtilis recombinant strains expressing Cyt2Ba (PB7231, PB7266 + IPTG), Cry11Aa (PB7233, PB7265) or both toxins (PB7240 and PB7265 + IPTG) compared to control strains PB7229 and PB7266. All strains were grown in 2xSG medium. Data from a single representative experiments are reported. [file MBT2-13-1972-s007.tiff]
