# Supplementary material for: Bacillus subtilis as a host for mosquitocidal toxins production
Source: Microb Biotechnol. 2020 Aug 30;13(6):1972–82. doi: 10.1111/1751-7915.13648 (PMC7533320; doi:10.1111/1751-7915.13648)
Supplement: Supplementary file 8 — Table S1. Primers used for cloning. Restriction sites are shown in capital letters. [file MBT2-13-1972-s008.docx]

**Table S1.** Primers used for cloning. Restriction sites are shown in capital letters

| Name | Sequence |
| --- | --- |
| yhfORev | ccggtatcctcttcatagccgttt |
| aprEcry11AaR | atctaaagaactatcttccattctttaccctctccttttaaaaaaattc |
| aprEcry11AaF | gaatttttttaaaaggagagggtaaagaatggaagatagttctttagat |
| cry11AaDwExt | cccccatactaatgtttccagtgca |
| oGB1 | gcctatgaattctccattttcttc |
| pBt021DwBam | cagaGGATCCggttaaacgttccgattaag |
| oGB1SphI | ggccatGCATGCgcctatgaattctccattttcttc |
| aprERXbaI | ccacaattttttgcttctcacTCTAGAccctctccttt |
| cry11Up | gcgcGTCGACcaataaaaggtggaatgaattatatgg |
| cry11Dw | gacaGCATGCctcgtatactctgtttcctc |
| cyt2BAUp | gacaGTCGACctttagaccgtacgttcgga |
| cyt2BADw | gacaGCATGCgagcgcgtttgaaaagaag |
| cyt2d | aatacatttcaaggagcta |
| cyt2r | tttcattttaacttcatatc |
| cyt1AAUp | gcgcGTCGACcttaaggagttgtttatttatgg |
| cyt1AAUp1 | gacaGTCGACcaggcatctttcgaactatagc |
| pBt020Dw | gacaGCATGCcgaaaaatgtggatgtgtgaag |
| pBt021Dw | gacaGCATGCggttaaacgttccgattaag |
| cyt2BAUp1 | gacaGTCGACcgttgtcgaagaaaggattc |
| gral-cyt1d | aacccctcaatcaacagcaagg |
| gral-cyt1r | ggtacacaatacataacgccacc |
