# Supplementary material for: Bacillus subtilis as a host for mosquitocidal toxins production
Source: Microb Biotechnol. 2020 Aug 30;13(6):1972–82. doi: 10.1111/1751-7915.13648 (PMC7533320; doi:10.1111/1751-7915.13648)
Supplement: Supplementary file 9 — Table S2. Plasmids used in this work. [file MBT2-13-1972-s009.docx]

**Table S2** Plasmids used in this work

| Plasmid | Description | Reference |
| --- | --- | --- |
| pDR111 | Derivative of the *Pspac-hy* plasmid pJQ43 (Quisel, et al., 2001). It carries a spectinomycin resistance cassette, a multiple cloning site (MCS) downstream of the IPTG-inducible *Phyperspank* promoter, and the gene encoding the LacI repressor between the two arms of the *Bacillus subtilis amyE* gene*.* Thanks to an extra *lacO* binding site, it ensures better repression in the absence of the IPTG inducer (Britton, et al., 2002). | (Ben-Yehuda, et al., 2003)  Kind gift of D. Rudner, Harvard Medical School  BGSCID*: ECE312 |
| pBS19 | Multi-copy plasmid derivative of pBS42 (Band and Henner, 1984). It contains the pUC18 multiple cloning site and the *cat* resistance gene from pC194 | (Perego and Hoch, 1991)  GenBank: JA139054.1 |
| pJM113 | *B. subtilis* integrative plasmid carrying the MCS of pUC19 and the *Streptococcus faecalis* kanamycin resistance gene (Accession: V01547.1). | (Perego, 1993) |
| pDR111-*cry11Aa-p20* | Derivative of plasmid pDR111 carrying the Bti *cry11-p20* genes between the *SalI-SphI* restriction sites. | This work |
| pDR111-*cyt2BA* | Derivative of plasmid pDR111 carrying the Bti *cyt2BA* gene between the SalI-SphI restriction sites | This work |
| pBS19-P*cyt1Aa-p21* | Derivative of plasmid pBS19 carrying the Bti *cyt1Aa-p21* genes under the control of *cyt1Aa* promoter between the SalI-SphI restriction enzymes | This work |
| pBS19-P*cyt2BA* | Derivative of plasmid pBS19 carrying the Bti *cyt2BA* gene under the control of its own promoter between the SalI-SphI restriction enzymes | This work |
| pBG105 | Derivative of pJM113 carrying the *PaprE-cry11Aa-p20* construct cloned between XbaI and BamHI restriction sites | This work |
| pBG109 | Derivative of pJM113 carrying the  *PaprE* fragment cloned between SphI and XbaI restriction sites. | This work |

- BGSCID: Bacillus Genetic Stock Center catalogue number ID

**References**

Band, L., and Henner, D.J. (1984) *Bacillus subtilis* requires a "stringent" Shine-Dalgarno region for gene expression. *DNA* **3**: 17-21.

Ben-Yehuda, S., Rudner, D.Z., and Losick, R. (2003) RacA, a bacterial protein that anchors chromosomes to the cell poles. *Science* **299**: 532-536.

Britton, R.A., Eichenberger, P., Gonzalez-Pastor, J.E., Fawcett, P., Monson, R., Losick, R., and Grossman, A.D. (2002) Genome-wide analysis of the stationary-phase sigma factor (sigma-H) regulon of *Bacillus subtilis*. *J Bacteriol* **184**: 4881-4890.

Perego, M. (1993) Integrational vectors for genetic manipulation in *Bacillus subtilis*. In: *Bacillus subtilis and other Gram-positive bacteria.* Sonenshein, A.L., Hoch, J.A., and Losick, R. (eds). Washington D.C.: American Society of Microbiology, pp. 615-624.

Perego, M., and Hoch, J.A. (1991) Negative regulation of *Bacillus subtilis* sporulation by the Spo0E gene product. *J Bacteriol* **173**: 2514-2520.

Quisel, J.D., Burkholder, W.F., and Grossman, A.D. (2001) *In vivo* effects of sporulation kinases on mutant Spo0A proteins in *Bacillus subtilis*. *J Bacteriol* **183**: 6573-6578.
