# Supplementary material for: Bacillus subtilis as a host for mosquitocidal toxins production
Source: Microb Biotechnol. 2020 Aug 30;13(6):1972–82. doi: 10.1111/1751-7915.13648 (PMC7533320; doi:10.1111/1751-7915.13648)
Supplement: Supplementary file 10 — Table S3. Titer of cells and spores (cfu ml−1) in spore‐parasporal bodies mixtures resuspended at the concentration of 100 mg l−1. Results refer to the following strains: PB7229 (negative control, pBS19) and PB7230 (pBS19‐Pcyt1Aa‐p21), collected at 24, 48, 72 h after the beginning of the stationary phase (T24, T48, T72). Results are means of three replicas ± SD. [file MBT2-13-1972-s010.docx]

**Table S3** Titer of cells and spores (cfu/ml) in spore-parasporal bodies mixtures resuspended at the concentration of 100 mg/L. Results refer to the following strains: PB7229 (negative control, pBS19) and PB7230 (pBS19-*Pcyt1Aa-p21*)*,* collected at 24, 48, 72 hours after the beginning of the stationary phase (T24, T48, T72). Results are means of three replicas ± SD

|  | **Time of collection** | **% sporulation ± S.D.** | **Average of spores/ml ± S.D. after 10 min at 80 C** | **Average of cfu/ml ± S.D.** |
| --- | --- | --- | --- | --- |
|  |  |  |  |  |
| **PB7229** | T24 | 46% ± 19% | 6,90E+05 ± 8,57E+04 | 1,65E+06 ± 6,13E+05 |
|  | T48 | 53% ±21% | 1,55E+06 ± 1,74E+06 | 2,92E+06 ± 2,43E+06 |
|  | T72 | 67% ±17% | 5,69E+05 ± 2,60E+05 | 9,32E+05 ± 5,72E+05 |
| **PB7230** | T24 | 1,1% ± 1% | 3,26E+02 ± 3,18E+01 | 6,22E+04 ± 6,20E+04 |
|  | T48 | 67% ± 3% | 1,71E+02 ± 5,01E+01 | 2,59E+02 ± 8,62E+01 |
|  | T72 | 64% ± 18% | 2,56E+02 ± 2,61E+02 | 8,14E+02 ± 1,13E+03 |
