# Supplementary material for: Bacillus subtilis as a host for mosquitocidal toxins production
Source: Microb Biotechnol. 2020 Aug 30;13(6):1972–82. doi: 10.1111/1751-7915.13648 (PMC7533320; doi:10.1111/1751-7915.13648)
Supplement: Supplementary file 11 — Supplementary Information. Genetic techniques employed in the study and detailed description of the preparation of the B. subtilis strains expressing δ‐endotoxins. [file MBT2-13-1972-s011.docx]

**Supplementary Information**

**Materials and Methods**

**Genetic techniques**

A slightly modified version of Kunst’s protocol (Kunst and Rapoport, 1995) was used to prepare competent cells of *B. subtilis* wild-type strains.

In *B. subtilis* strains harboring the *PxylA-comK* construct (PB7007 and in its derivatives), competence was induced with 0.3 % xylose at OD600 = 1.

In both techniques, the transforming plasmid DNA was previously amplified with the Illustra TempliPhi Amplification Kit (GE Healthcare Lifescience).

*E. coli* DH5α transformation was performed according to standard protocols (Orkin, 1990).

**Construction of *B. subtilis* δ-endotoxins expression strains**

In order to place the *cry11Aa* gene under the control of the IPTG-inducible *Phyperspank* promoter, a 2124 bp DNA fragment corresponding to *cry11Aa* coding sequence was amplified with primers cry11Up and cry11Dw (Table S1) using *B. thuringiensis* 4Q1 pBtoxis DNA (NCBI Reference Sequence: NC_010076.1) as template. The purified DNA (QIAquick PCR Purification Kit, Qiagen) was cloned in the *amyE* integrative plasmid pDR111 using SalI and SphI restriction sites. The resulting plasmid (pDR111-*cry11Aa*) was *in vitro* amplified by rolling circle amplification (Illustra TempliPhi Amplification Kit, GE Healthcare Lifescience) and used to transform *B. subtilis* PB1831 competent cells, creating strain PB7223. As a control, PB1831 was transformed with empty pDR111 to obtain PB7222.

All other pDR111 derivative plasmids constructed in this work (Table S2) were obtained in the same way, by cloning the genes of interest between the SalI-SphI restriction sites, downstream of the IPTG-inducible *Phyperspank* promoter.

To prepare plasmid pDR111-*cry11Aa-p20*, the 2819 bp region encoding *cry11Aa* and *p20* was amplified with primers cry11Up and pBt021Dw (Table S1). The resulting vector was used to transform *B. subtilis* strain PB1831, thus obtaining PB7226. The same *Phyperspank-cry11Aa-p20* construct was also introduced in a *degU(hy)* background. To this purpose, the chromosomal DNA of strain PB7226 was used to transform *B. subtilis* strain PB7007 (*degU(hy)*). Transformants carrying the P*hyspank*-*cry11Aa-p20* construct integrated at the *amyE* locus were selected by spectinomycin resistance. Clones in which integration had occurred by a double cross-over, homologous recombination event at the *amyE* locus (Δ*amyE*) were identified by performing an amylase assay on LB plates containing insoluble starch. The resulting strain was named PB7247. A control strain (PB7246) was prepared by transforming PB7007 with PB7222 (Δ*amyE*::*spc*) chromosomal DNA.

To construct pDR111-*cyt1Aa-p21*, the genomic region of 938 bp encoding *cyt1Aa* and *p21* was amplified with the primers cyt1AAUp and pBt020Dw. The obtained plasmid was used to transform *B. subtilis* PB1831 strain, thus obtaining strain PB7232.

The pDR111-*cyt2Ba* vector was built by amplifying the 980 bp gene *cyt2BA* with primers cyt2BAUp and cyt2BADw and was used to transform *B. subtilis* strain PB1831, giving rise to PB7225.

In order to construct an auto-inducible *B. subtilis* strain expressing the *cry11Aa-p20 B. thuringiensis israelensis* genes under the control of the *B. subtilis* *aprE* promoter, an 868-bp DNA fragment encompassing the entire transcriptional control region (TCR) of *aprE* was amplified by PCR with primers yhfORev and aprEcry11AaR using the chromosomal DNA of the wild-type *B. subtilis* strain PB168 (NCBI Reference Sequence: NC_000964.3) as template. The 2959 bp pBtoxis region encompassing the coding sequences of *cry11Aa* and *p20* was amplified by using primers aprEcry11AaF and cry11AaDwExt. The obtained PCR fragments were purified by gel extraction (Nucleospin Gel and PCR Clean-up kit, Macherey-Nagel) and used as templates in a fusion PCR performed using the primers oGB1 and pBt021DwBam. The fusion product of 3400 bp was digested with XbaI and BamHI (FastDigest, ThermoFisher Scientific) and inserted into plasmid pJM113, thus obtaining pBG105. To create the control vector, the DNA of *B. subtilis* PB168 was used as template for the amplification of the entire transcriptional control region of *aprE* using primers oGB1SphI and aprERXbaI. The obtained fragment was SphI-XbaI digested and cloned into pJM113, the resulting vector was named pBG109. The two pJM113 derivative vectors were amplified *in vitro* by rolling circle replication and used to transform xylose induced competent cells of the *degU32(hy)* mutant strain PB7007. The resulting strain carrying the pBG105 plasmid integrated by single crossover at the *aprE* locus was named PB7241. The control strain obtained by transformation of PB7007 with pBG109 was named PB7242.

To co-express constructs *Phyperspank-cyt2Ba* and *PaprE-cry11Aa-p20* in a *degU(hy)* background, the chromosomal DNA of strain PB7225 (Δ*amyE*::*Physpank-cyt2Ba*) was used to transform strains PB7241 (Δ*aprE*::*PaprE-cry11Aa-*p20) and PB7242 (Δ*aprE*::*spc*). Transformants were selected on LB plates supplemented with spectinomycin 200 µg/ml. Stable integration of the *Physpank-cyt2Ba* construct by double-crossover, homologous recombination at the *amyE* locus of the recipient strains was verified by amylase assay performed on LB plates containing 2% insoluble starch. Clones not producing starch degradation halos (Δ*amyE*) were selected and named PB7265 and PB7266.

The *B. thuringiensis* *cyt1Aa-p21* cluster was cloned under the control of *cyt1Aa* promoter in the *B. subtilis* multicopy replicative plasmid pBS19. To this purpose, the 1224 bp region of interest was amplified with primers cyt1AAUp1 and pBt020Dw and cloned into pBS19, by making use of SalI-SphI restriction sites. Since plasmid pBS19 has no origin of replication for *E. coli*, the ligation product pBS19-*Pcyt1Aa-p21* was amplified by TempliPhi reaction and transformed directly into *B. subtilis* PB1831 competent cells. The transformed colonies were selected directly in *B. subtilis* for the acquisition of chloramphenicol resistance and the correct clones carrying the recombinant vector were identified by colony PCR with primers gral-cyt1d and gral-cyt1r. The resulting strain was named PB7230. The parental vector pBS19 was used to transform *B. subtilis* PB1831 to create the control strain PB7229.

Finally, *cyt2BA* gene was cloned under the control of its own promoter. The 1247 bp region of *B. thuringiensis israelensis* 4Q1 pBtoxis plasmid, encompassing the *cyt2BA* gene with its promoter, was amplified with primers cyt2BAUp1 and cyt2BADw, digested with SalI-SphI restriction enzymes, and cloned inside the multicopy replicative vector pBS19, thus obtaining pBS19-*Pcyt2BA*. The pBS19-*Pcyt2Ba* ligation product was amplified through rolling circle amplification (Illustra TempliPhi Amplification Kit, GE Healthcare Lifescience) and used to transform directly *B. subtilis* PB1831 and PB7226 competent cells. The correct clones were identified through colony PCR with the primers pair cyt2d/cyt2r, giving rise to PB7231 and PB7240, respectively. The parental vector pBS19 was used to transform *B. subtilis* PB7226 to create the control strain PB7233.

**Supplementary Information References**

Kunst, F., and Rapoport, G. (1995) Salt stress is an environmental signal affecting degradative enzyme-synthesis in *Bacillus subtilis*. *J Bacteriol* **177**: 2403-2407.
